# The role of the gut microbiome in paediatric irritable bowel syndrome

**DOI:** 10.3934/microbiol.2022030

**Published:** 2022-11-22

**Authors:** Alexandra S McHarg, Steven Leach

**Affiliations:** 1 School of Women's and Children's Health, Faculty of Medicine, University of New South Wales, Sydney, NSW, Australia; 2 Westfield Research Laboratories, Sydney Children's Hospital, Randwick, NSW, Australia

**Keywords:** irritable bowel syndrome, gut microbiome, dysbiosis, child, diet, low-FODMAP

## Abstract

Irritable bowel syndrome (IBS) is a common and disabling condition in children. The pathophysiology of IBS is thought to be multifactorial but remains incompletely understood. There is growing evidence implicating the gut microbiome in IBS. Intestinal dysbiosis has been demonstrated in paediatric IBS cohorts; however, no uniform or consistent pattern has been identified. The exact mechanisms by which this dysbiosis contributes to IBS symptoms remain unknown. Available evidence suggests the imbalance produces a functional dysbiosis, with altered production of gases and metabolites that interact with the intestinal wall to cause symptoms, and enrichment or depletion of certain metabolic pathways. Additional hypothesised mechanisms include increased intestinal permeability, visceral hypersensitivity and altered gastrointestinal motility; however, these remain speculative in paediatric patients, with studies limited to animal models and adult populations. Interaction between dietary components and intestinal microbiota, particularly with fermentable oligosaccharides, disaccharides, monosaccharides and polyols (FODMAPs), has drawn increasing attention. FODMAPs have been found to trigger and worsen IBS symptoms. This is thought to be related to products of their fermentation by a dysbiotic microbial population, although this remains to be proven. A low-FODMAP diet has shown promising success in ameliorating symptoms in some but not all patients. There remains much to be discovered about the role of the dysbiotic microbiome in paediatric IBS.

## 1. Introduction

Irritable bowel syndrome (IBS) is a chronic functional gastrointestinal (GI) disorder, characterised by abdominal pain and bloating associated with alterations in stool consistency and frequency, with no evidence of any organic bowel pathology [1]. It can be classified into subtypes according to the predominant stool form: constipation (IBS-C), diarrhoea (IBS-D) or mixed constipation and diarrhoea (IBS-M), pursuant to the Rome IV criteria [1]. The prevalence of IBS in children varies, with reported rates of up to 19.8% in general population studies [2]. IBS is a disabling condition, causing reduced health-related quality of life [3], poorer educational outcomes [4] and increased stress and anxiety [5]. Current understanding of IBS pathophysiology is incomplete; however, many factors have been implicated, including visceral hypersensitivity, gastrointestinal dysmotility, mucosal inflammation, increased intestinal permeability, altered host immune responses, bile acid malabsorption, psychological disturbances and genetic factors [6]–[7]. There is also strong and growing evidence implicating the gut microbiome in IBS pathophysiology. This is exemplified in the increased risk of developing IBS following gastroenteritis [8], following a course of antibiotics [9] and with co-existing small intestinal bacterial overgrowth [10]—all processes that cause a disturbance in the gut microbiota. This article will review the current evidence regarding the composition and role of the intestinal microbiota in paediatric IBS and review the interactions between microbes and diet, specifically fermentable carbohydrates, that may be contributing to the development of IBS symptoms.

## 2. A dysbiotic microbiome in paediatric IBS

The gut microbiome is the community of microbes and their collective genomes that reside in the lower GI tract of humans. It is composed largely of bacteria, as well as of archaea, eukaryotes and viruses [11]. In healthy individuals, the gut microbiome exists in a symbiotic relationship with the host—microbes make available nutrients and energy from food that the host was initially unable to utilise, in return for a stable habitat [12]. An alteration or imbalance in these microbial communities disrupts this symbiotic relationship and is termed a ‘dysbiosis.’

It is well established that the gut microbiomes of adults with IBS are dysbiotic compared to healthy controls (HC), as consistently demonstrated in numerous cross-sectional studies [13]–[20]. The development of technologies such as 16S ribosomal-RNA (rRNA) sequencing have enabled detailed metagenomic analysis of microbial communities down to the species level. Despite this, this expanding body of studies report inconsistent and conflicting trends and lack an overall consensus. A uniform pattern of dysbiosis in IBS remains to be identified.

Our understanding of the microbial communities in paediatric IBS is even more uncertain—limited to date to only three small cross-sectional studies [21]–[23]. In these studies, dysbiosis in paediatric IBS was characterised by increased phylogenetic diversity compared to HC and shifts in the abundance of many microbes at different taxonomic levels, as demonstrated in Table 1. The only consistent finding across these results is enrichment of class Gammaproteobacteria in IBS microbial communities, a promising indication of its involvement in IBS pathophysiology. However, the remainder and vast majority of findings are poorly replicated between these paediatric populations. Of particular note, there is conflict in the reported abundances of *Bacteroides* species. The inconsistencies and lack of consensus between these studies could be explained by several differences in study design, including varied definitions of IBS, evaluation of disparate or mixed IBS subtypes and differences in molecular sequencing techniques, as detailed in Table 2. These all act as potential confounders: Different subtypes of IBS are known to demonstrate distinct microbial populations [24], varied inclusion criteria decrease comparability of results, microbiome composition changes with age [25], different gene amplification primers and sequencing techniques may result in variable coverage of microbial environments [26], and dietary variation, not consistently controlled across studies, is known to influence the gut microbiota [27]. The divergent findings to date demonstrate that microbiome dysbiosis in paediatric IBS remains insufficiently characterised.

Compared to reports from adult cohorts, these paediatric findings show both similarities and differences. Enrichment of Gammaproteobacteria has been a reoccurring observation in IBS adults [16],[28],[29], comparable to these paediatric findings. Adult studies have also similarly found mixed results with *Bacteroides* species, with both increased [20],[24] and decreased [15],[17] abundances observed. Surprisingly, significant disparities in the dominant bacterial phyla Firmicutes and Bacteroidetes have not been observed among paediatric IBS cohorts, despite strong but conflicting differences in adult IBS cohorts [15],[17]–[19]. Overall, this suggests that the dysbiosis in paediatric IBS microbiomes is distinct from the dysbiosis observed in adults. However, this hypothesis cannot be validated due to an absence of studies directly comparing adults and children with IBS.

Further, caution should be exercised when interpreting and applying these findings to other paediatric IBS populations, considering the following threats to their external validity. Firstly, relatively small sample sizes of 22–23 IBS and 22 HC in all three studies limit their statistical power. Secondly, studies were conducted on American populations, and since geographical location has been demonstrated to influence the gut microbiome [30], their universal applicability is potentially limited. Finally, there have not been any further studies conducted in paediatric IBS cohorts to confirm or challenge these findings in order to draw meaningful and reliable conclusions. Further investigation is warranted, with larger cohorts and consistent inclusion criteria, to define a clearer picture of the gut microbial communities in paediatric IBS.

**Table 1.** Bacterial community compositional differences observed in the paediatric IBS microbiome in cross-sectional studies according to taxonomic level.

Research paper	Class	Family	Genus	Species
*Saulnier et al*. [21]	↑Gammaproteobacteria	↑ Clostridiaceae ↑ Enterobacteriaceae ↑ Prevotellaceae	↑ *Haemophilus* ↑ *Dorea* ↑ *Ruminococcus* ↓ *Eubacterium* ↓ *Anaerovorax*	↑ *Haemophilus parainfluenzae* ↑ novel *Ruminococcus* species ↓ several *Bacteroides* species
*Rigsbee et al*. [22]			↑ *Enterobacter* ↑ *Lactobacillus* ↑ *Megasphaera* ↑ *Oxalobacter* ↑ *Parasporobacterium* ↑ *Prevotella* ↑ *Raoultella* ↑ *Veillonella* ↓ *Bifidobacterium* ↓ *Dehalobacter* ↓ *Fusibacter* ↓ *Oxobacter*	↑ *Bacteroides eggerthii*
*Hollister et al*. [23]	↑ Gammaproteobacteria	↑ unclassified Clostridiales family	↑ *Flavonifractor* ↑ *Lachnospiraceae bacterium 7_1_58FAA*	↑ *Flavonifractor plautii*

**Table 2.** Differences in study design.

Research paper	IBS inclusion criteria	IBS subtypes studied	Molecular sequencing technique	Analytical tests	Consideration of dietary variation
*Saulnier et al*. [21]	Age 7–12 Paediatric Rome III IBS criteria Gastrointestinal Symptom Rating Scale-IBS questionnaire	IBS-C IBS-D IBS-M	16s ribosomal RNA sequencing with forward primer 27F and reverse primer 534R. V1–V3 and V3–V5 regions amplified by PCR.	Wilcoxon rank sum tests	No control prior to sample collection
*Rigsbee et al*. [22]	Age 8–18 Paediatric Rome II IBS Criteria	IBS-D only	16s ribosomal RNA sequencing with forward primer 27F and reverse primer 533R. V1–V3 region amplified by PCR.	Mann-Whitney U tests	Children on restricted diets excluded No control prior to sample collection
*Hollister et al*. [23]	Age 7–12 Paediatric Rome III questionnaire ICD-9*	IBS-C IBS-D IBS-M IBS-U	Whole genome shotgun sequencing	Wilcoxon rank sum tests with Benjamini-Hochberg corrections	24-hour food diaries completed by a subset of participants

*ICD-9 = International Classification of Diseases, Ninth Revision

## 3. A pathogenic role of gut microbiota?

**Figure 1.**
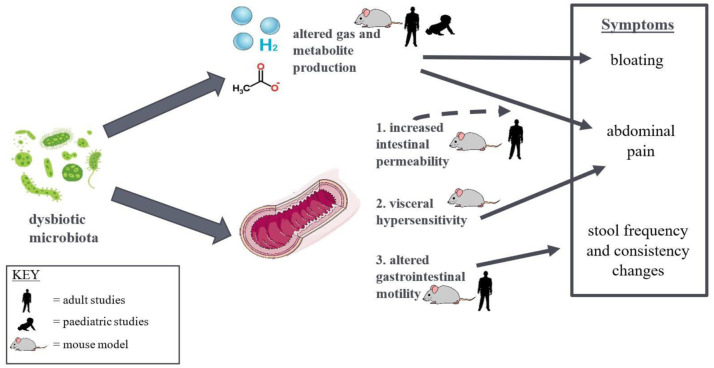
Current hypotheses with supportive evidence on the role of dysbiotic microbes in IBS pathophysiology and symptom generation.

Recently, there has been a shift towards identifying the functional consequences of this observed microbial dysbiosis and the mechanisms by which it contributes to IBS symptoms. Associations between microbe abundances and symptoms have been described, including *Ruminococcus* with increased abdominal pain frequency in both children [21] and adults [19]. However, due to cross-sectional study design, it cannot be established whether these are correlation or causation relationships. Nonetheless, it is currently thought that the dysbiotic microbiota produce imbalanced levels of metabolites and gases—termed a functional dysbiosis—which interact with the intestinal wall and environment to trigger symptoms. Figure 1 depicts this hypothesis and the evidence supporting it. Evidence in paediatric IBS comes firstly from Shankar et al. [31], who, by adding metabolomic profiling to the samples from the Rigsbee et al. [22] study, demonstrated higher levels of formate, glucose, lactate and several amino acids, and lower levels of pyruvate, in the IBS group. These levels were suggested to represent reduced methane production, incomplete anaerobic fermentation and increased proteolysis, respectively [31]. The latest paediatric IBS microbiome study conducted by Hollister et al. [23] also investigated the expression of metabolites and enzymes. They found significant enrichment of sterols, steroids, bile acids and products of phenylalanine and tyrosine degradation, and they found positive correlations between their concentrations and the frequency and severity of abdominal pain. Additionally, they reported relative enrichment of metabolic pathways and enzymatic reactions related to amino acid metabolism, phospholipid biosynthesis and vitamin biosynthesis and relative depletion of those related to carbohydrate metabolism. Table 3 summaries these metabolic differences identified in IBS cohorts.

Studies in adults have also found altered levels of metabolite production and metabolic pathways. A cross-sectional study of 26 adult IBS patients found higher levels of total organic acids and two short chain fatty acids (SCFAs), acetic acid and propionic acid, and a correlation between these high levels and increased severity of abdominal pain and bloating [32]. A larger study in IBS-D and IBS-M adults (n = 56) identified a subset of IBS patients with dysbiotic microbiomes, enriched in Bacteriodetes whilst depleted in Firmicutes, that demonstrated enrichment of metabolic pathways related to amino acid and carbohydrate metabolism [33]. A smaller cross-sectional study of IBS-C females (n = 14) observed greater production of sulphides and hydrogen, and reduced production of butyrate, compared to healthy subjects [16], also reflecting altered intestinal fermentation. This study group suggested that accumulated hydrogen may be responsible for gas-related symptoms of bloating and flatus, and toxic sulphides may play a key role in visceral hypersensitivity leading to abdominal pain [16]. A subsequent randomised controlled trial (RCT) in germ-free rats provided evidence to support this [34]. In this study, inoculating germ-free rats with faecal microbiota from IBS patients induced visceral hypersensitivity, demonstrating a causal relationship between microbiota and IBS symptoms. Furthermore, these rats produced increased hydrogen gas and sulphides compared to controls inoculated with healthy samples [34]. This mirrors the results from Chassard and colleagues [16] and supports the role of sulphides in generating the visceral hypersensitivity that is responsible for abdominal pain in IBS.

It is also thought that microbial metabolites generate IBS symptoms by traveling through an impaired epithelial barrier and provoking abnormal immune- and neuro-responses in the gut wall [15],[19]. SCFAs, metabolites produced by the fermentation of complex carbohydrates, are known to regulate intestinal permeability. For example, butyrate, the primary energy source for colonocytes, enhances the intestinal barrier by facilitating the assembly of tight junctions [35]. Impaired epithelial integrity has been previously described in some IBS patients [36]. In a more recent RCT, increased paracellular permeability was observed in germ-free mice after receiving IBS-D microbiota [37], providing evidence to support a pathogenic role of dysbiotic microbes. Additionally, these mice demonstrated faster gastrointestinal transit. This confirms an earlier cross-sectional study involving 114 adult IBS patients and 33 healthy controls that found an association between increased production of SCFAs and increased gastrointestinal motility, faster transit times and increased stool frequency [38]. These studies provide evidence to support the hypothesis that microbes and their metabolites can provoke abnormal neuro-responses in the intestine, affecting colonic motility that manifests in altered bowel habit. However, a lack of similar studies in paediatric IBS means that these causal links found in adult IBS cannot be confirmed to be involved in the paediatric form of the condition. Additionally, many of these findings linking the gut microbiota to symptom generation are in animal models and have not been replicated in human studies to confirm the relationships. One must therefore be hesitant to accept these results, especially in their application to children. The defined role of the dysbiotic microbes and their metabolites in paediatric IBS remains speculative. Further research modelling metabolite production and the functional capabilities of paediatric IBS microbiota would allow determination of the causal relationship between dysbiotic microbiota and IBS symptoms, which would certainly be the more clinically relevant information and would lead to a greater capacity to develop targeted treatments.

**Table 3.** Bacterial community functional differences observed in IBS microbiomes (paediatric and adult).

Research paper	Metabolites		Metabolic pathways	
	Increased	Decreased	Enriched	Depleted
*Shankar et al*. [31] (paediatric)	formate, glucose, lactate	pyruvate		
*Hollister et al*. [23] (paediatric)	sterols, steroids, bile acids, products of phenylalanine and tyrosine degradation		amino acid metabolism, phospholipid biosynthesis, vitamin biosynthesis	carbohydrate metabolism
*Tana et al*. [32] (adult)	acetic acid, propionate acid			
*Chassard et al*. [16] (adult)	sulphides, hydrogen	butyrate		
*Vervier et al*. [33] (adult)			amino acid biosynthesis (tryptophan, threonine, histidine), carbohydrate metabolism (lactose, fructose and trehalose metabolism; butyrate and propionate biosynthesis)	

## 4. Fermentable carbohydrates and the IBS microbiome

Another speculated role by which dysbiotic microbes trigger IBS symptoms is through interacting with dietary nutrients. Abdominal pain and bloating are reportedly triggered by specific foods in 63–90% of patients [39]. The intestinal microbiota plays an integral role in the breakdown of dietary components within the colon, achieved through fermentation. This process produces gases, including hydrogen and methane, and SCFAs, predominantly acetate, butyrate and propionate [38]. In recent years, there has been growing interest in the role of undigestible, poorly absorbed, fermentable carbohydrates, termed fermentable oligosaccharides, disaccharides, monosaccharides and polyols (FODMAPs). As FODMAPs are poorly absorbed, they have an osmotic effect on the small bowel [40]–[42] and are subsequently fermented by colonic bacteria, resulting in increased production of gas [41],[43]. This increased fluid load and gas causes intestinal distension. In a mouse model, fermentable carbohydrates also increased gastrointestinal motility, shortening transit time [44]. These are normal physiological phenomena that are tolerated by a healthy colon [41], with the only reported symptom of slightly increased flatulence [45]. However, in adult IBS patients, FODMAPs significantly worsen gastrointestinal symptoms of abdominal pain, bloating and flatulence and increase lethargy, as demonstrated in a randomised crossover trial [45]. A more recent randomised crossover trial confirmed this, finding that IBS patients experience increased frequency and severity of gastrointestinal symptoms following fermentable carbohydrate ingestion compared to control subjects [42]. This has also been demonstrated in children with IBS, through a 72-hr fructan challenge (fructan being an example of a FODMAP), which demonstrated worsening of abdominal pain frequency, bloating and hydrogen production compared to placebo in a randomised, double-blind, crossover trial [46].

A proposed reason for why FODMAPs trigger and exacerbate symptoms in IBS, but minimally in healthy subjects, is that the fermentation of FODMAPs by dysbiotic IBS microbiota produces excessive amounts of gas compared to healthy microbiota, which further distends the colon, resulting in abdominal pain, bloating and flatulence. This has been supported by the observed depletion of metabolic pathways related to carbohydrate metabolism in paediatric IBS microbiomes [23].

Carbohydrates remaining poorly digested due to these depleted pathways would progress to the colon, where their fermentation may produce excessive amounts of gas and other irritating metabolites—which could be key to symptom generation. However, other studies in the literature present conflicting evidence. Whilst Ong et al. [45] found that IBS patients produced increased gas on a high-FODMAP diet compared to healthy subjects, interestingly Major et al. [42] found comparable levels of increased colonic volume and gas production between IBS and healthy subjects. This apparent discrepancy could be explained by the differing techniques used to measure colonic gas production—breath hydrogen and MRI imaging, respectively—rendering these results difficult to compare. It could also indicate that alternative mechanisms contribute to symptom genesis, particularly how visceral hypersensitivity may trigger pain and bloating in response to the stimulus of intestinal distension, or the effect of bacterial metabolites on the enteric nervous system. It has also been suggested that motility responses secondary to luminal distension, coupled with increased fluid, may contribute to loose stools in the IBS-D subtype [45]. However, the exact role of the dysbiotic microbiota in the fermentation of dietary components such as FODMAPs to produce IBS symptoms is unclear.

Further evidence pointing towards the role of fermentable carbohydrates in triggering IBS symptoms is the building evidence-base for the efficacy of a low-FODMAP diet. The most significant study to date was a randomised, controlled, single-blind, crossover trial, which demonstrated that a low-FODMAP diet, compared to a typical Australian diet, produced significant overall improvement in gastrointestinal symptoms, including bloating, abdominal pain and flatulence, in 70% of IBS adults [47]. Subsequent studies have supported this, demonstrating significant symptom improvement on a low-FODMAP diet in 50–85% of adult patients [48]–[53], including demonstrating superiority over placebo [50] and standard IBS dietary guidelines [49]. This broad range of responsiveness could be explained by different lengths of dietary intervention, different efficacy end points and different methods of determining compliance between studies, and whether meals were provided to participants compared to only receiving dietician counselling. The efficacy of a low-FODMAP diet in paediatric IBS has not been as extensively investigated. Only a single trial has been conducted [54], which involved 33 children in a double-blind, randomised, cross-over design comparing a low-FODMAP diet to a traditional American childhood diet (TACD). The low-FODMAP diet significantly reduced the number and severity of abdominal pain episodes and total gastrointestinal symptom score compared to TACD and baseline [54]. However, only 24% of children were characterised as ‘responders’, a significantly lower proportion than has been reported in adult studies. This suggests that a low-FODMAP diet may be less effective in paediatric IBS, or effective in a smaller cohort of children. However, the dietary intervention period was only 48 hours in this study, much shorter than the design of the many adult studies; therefore, a longer dietary period may be needed to see similar effects. Since only one study has been conducted in paediatric populations, further research is needed to clarify the efficacy and mechanism of a low-FODMAP diet in paediatric IBS.

Subsequently, it has been proposed that certain gut microbes, and the presence or absence of gut dysbiosis, may be central to the efficacy of a low-FODMAP diet. This concept first developed when Chumpitazi and colleagues [54] found that ‘responders’ to a low-FODMAP diet were enriched at baseline in several bacterial taxa, compared to ‘non-responders.’ These included Bacteroidaceae, Erysipelotrichaceae and Ruminococcaceae families, *Dorea* genus and *Faecalibacterium prausnitzii* species. These bacteria have a larger number of carbohydrate-active enzymes and therefore have greater saccharolytic capacity [54], which suggests that carbohydrate-fermenting bacteria play a role in the success of the low-FODMAP diet. A similar study group compared the microbiomes of IBS children who were ‘fructan-sensitive’ vs ‘fructan-tolerant’, as defined by a ≥30% increase in abdominal pain frequency during a fructan diet [55]. They discovered distinct differences between fructan-sensitive and fructan-tolerant subjects' microbiomes (at baseline and during dietary interventions) in alpha diversity (lower in ‘fructan-sensitive’), beta diversity and taxonomic composition. Specifically, those ‘fructan-sensitive’ were enriched in the genus *Holdermania* at baseline, a microbe that can metabolise simple sugars but not complex carbohydrates, whilst those ‘fructan-tolerant’ were enriched in multiple genera from the class *Clostridia*, which have broad saccharolytic capacity and are thought to maintain gut homeostasis. However, no significant differences in fructan metabolism pathways were detected, a somewhat contradictory finding that demonstrates our understanding is incomplete and that the gut microbiome is incredibly complex. Studies in adults have also attempted to identify microbial signatures associated with response to a low-FODMAP diet [33],[56],[57], but there are no identifiable consistencies between these results.

Overall, these studies lend support to the gut microbiome being a mechanistic factor in FODMAP-induced IBS symptoms, but why some patients respond to a low-FODMAP diet and others do not remains incompletely understood. Further studies are needed to elucidate the exact mechanistic impact. Nonetheless, there is a promising indication that gut microbiome composition could be used as a tool in the future to identify IBS sufferers more likely to respond positively to a low-FODMAP diet, heading in the direction of personalised medicine.

## 5. Conclusion

It has been established that the gut microbiomes of both adult and paediatric IBS patients are dysbiotic. However, inconsistent microbiota have been implicated, and the patterns in IBS children appear different to those seen in IBS adults. Evidence suggests this dysbiosis has a pathogenic role in the development of IBS symptoms, through altered metabolite and gas levels interacting with the intestinal environment. However, our understanding of these mechanisms is poor and limited to animal models and adult studies. Their role in paediatric IBS remains speculative, and we should be hesitant to apply these directly to paediatric populations. Whilst the fermentation of dietary components, particularly of FODMAPs, has been demonstrated to trigger symptoms in IBS patients, the mechanisms responsible for this have been debated in the literature, and have not been as thoroughly investigated in paediatric IBS. The low-FODMAP diet has shown promising success in ameliorating symptoms but only in a small proportion of paediatric patients. Whilst particular bacterial taxa have been linked to the success of the low-FODMAP diet, these results are yet to be reproduced, and our understanding of how dysbiotic microbes interact with and process nutrients to cause symptoms is incomplete. A method of quantifying these interactions, through further investigation of the functional capabilities of the IBS microbiome and the interaction between microbes and nutrients, is needed and would open an avenue for refining and tailoring dietary intervention to individual patients with IBS.
